# Facile Synthesis of CoSe/Co_3_O_4_-CNTs/NF Composite Electrode for High-Performance Asymmetric Supercapacitor

**DOI:** 10.3390/ma15175841

**Published:** 2022-08-24

**Authors:** Ying Wang, Xiang Zheng, Xianjun Cao, Chengtao Yang, Qiang Zhao, Yongqi Zhang, Xinhui Xia

**Affiliations:** 1 Yangtze Delta Region Institute (Huzhou), University of Electronic Science and Technology of China, Huzhou 313001, China; 2 School of New Energy and Materials, Southwest Petroleum University, Chengdu 610500, China; 3 School of Materials and Energy, University of Electronic Science and Technology of China, Chengdu 610054, China; 4 Institute of Fundamental and Frontier Sciences, University of Electronic Science and Technology of China, Chengdu 610054, China

**Keywords:** CoSe/Co_3_O_4_-CNTs composite electrode, flame method, ultra-high specific capacitance, long cycle life, asymmetric supercapacitor

## Abstract

Electrode materials are key factors for supercapacitors to endow them with excellent electrochemical properties. Here, a novel hybrid structure of a CoSe/Co_3_O_4_-CNTs binder free composite electrode on nickel foam was prepared via a facile flame method, followed by an electrodeposition process. Benefitting from the synergetic effects of the multicomponent (with low resistances of 1.542 Ω cm^2^ and a moderate mesoporous size of 3.12 nm) and the enlarged specific surface area of the composite material (77.4 m^2^ g^−1^), the CoSe/Co_3_O_4_-CNTs composite electrode delivers a high specific capacitance of 2906 F g^−1^ at 5 mV s^−1^ with an excellent rate stability. The fabricated CoSe/Co3O4-CNTs/NF//AC ASC exhibits a high energy density of 43.4 Wh kg^−1^ at 0.8 kW kg^−1^ and a long cycle life (92.7% capacitance retention after 10,000 cycles).

## 1. Introduction

Highly efficient energy storage devices are one of the important means to satisfy the increasing energy demand. As an important piece of equipment for electrochemical energy storage, supercapacitors (SCs) have been a wide concern in the field of hybrid vehicles, consumer electronics, and industrial power management, because of their long maintenance-free lifetime, high power density, environmental protection, safety and reliability [1,2,3].

Depending on their various energy storage mechanisms, SCs can be compartmentalized into electric double layer capacitors (electrostatic storage charge), pseudocapacitors (reversible faradaic redox reaction storage charge) and asymmetric supercapacitors (ASCs). ASCs especially usually combine advantages of the two different types of electrodes, which can provide a high capacitance and a long cycle life [4]. As a main component of SCs, various electro-active materials have been explored, such as carbonaceous materials (carbon nanotubes [5,6,7] (CNTs), graphene [8,9] and active carbon [10]), transition metal-based materials (RuO_2_ [11], MnO_2_ [12], NiO [13], Co_3_O_4_ [14]), sulfides [15], metal selenides [16] and conducting polymers (PANI [17], PPy [18], PEDOT [19]).

Among them, transition metal selenides have been shown to possess higher electronic conductivity than oxides and sulfides and relatively high theoretical specific capacities, implying that transition metal selenides have great potential to be used as advanced electro-active materials for SCs [20,21]. However, the addition of binders usually decreases the electronic conductivity of the electrode, resulting in the deterioration of electrochemical performance. To solve this issue, some 3D porous metallic, such as Cu foam and nickel foam (NF), have been used as substrates to direct growth of electro-active materials to effectively promote the conductivity and electrochemical properties of the electrodes [22,23]. Meanwhile, the electrochemical properties of electro-active materials can be efficiently improved through fabrication of composite (hybrid) materials due to the complementary properties and unique synergistic effects of their multicomponent. For instance, hybrid structure CNT decorated NiSe_2_ nanosheets (NiSe_2_@CNT) [24], dual S, N-doped CoFe_2_O_4_@CNTs [25] and graphene nanosheet supported NiSe [26] have been proven to possess excellent electrochemical performance for supercapacitors. Recently, Zhu et al. [27] constructed a porous CNTs@NiCo-LDH nut-shell nanotube arrays for supercapacitor applications, which exhibited an improved mass transfer of ions and enhanced specific capacity.

Herein, we design a novel hybrid structure of CoSe/Co_3_O_4_-CNTs on NF via facile flame synthesis and followed by electrodeposition as a binder free electrode for SCs. Assisting with the synergetic effects of the multicomponent and low resistance, the as-obtained CoSe/Co_3_O_4_-CNTs/NF hybrid structure shows high specific capacitance (2906 F g*^−^*^1^, at 5 mV s*^−^*^1^) and rate performance (1362.8 F g*^−^*^1^, at 50 mV s*^−^*^1^). Furthermore, the constructed CoSe/Co_3_O_4_-CNTs/NF//AC ASC device delivers a high energy density of 43.4 W h kg^−1^ at 0.8 kW kg^−1^ with 92.7% capacitance retention after 10,000 cycles.

## 2. Experimental Section

### 2.1. Materials

Cobalt(III) acetylacetonate (Co(acac)_3_, Co(C_5_H_7_O_2_)_3_) was purchased from Alfa Aesar. Cobalt(II) chloride hexahydrate (CoCl_2_•6H_2_O, ≥99%) and lithium chloride monohydrate (LiCl•H_2_O, ≥95%) were obtained from Kelong Chemical Reagent Co. Ltd. (Chengdu, China) Selenium dioxide (SeO_2_, ≥99%) was acquired from Shanghai Macklin Biochemical Co., Ltd. (Shanghai, China). All chemical reagents were used directly.

### 2.2. The Synthesis of Co_3_O_4_-CNTs/NF

NF (with the thickness of 1.0 mm, surface density = 34.2 mg cm^−2^, pore per inch = 110) was immersed into a 3 M HCl solution for 1 min to remove the oxides and impurities on the surface firstly, then the precleaned NF was washed with acetone, ethanol, and DI water under the assistance of ultrasound. Finally, it was dried in a vacuum oven at 60 °C overnight.

Co_3_O_4_-CNTs loaded on the skeleton of NF was prepared via a facile flame method. Concisely, an ethanol solution of Co(acac)^3^ (with a concentration of 10 mg/mL) was prepared. Then, a piece of NF (with the area of 1 × 1 cm^2^) was immersed into the above solution for about 5 s to make the solution attach onto the skeleton of NF. After that, the NF was ignited and burned under ambient condition to gain the NF decorated by Co_3_O_4_-CNTs. To increase the mass loading of Co_3_O_4_-CNTs, the immersing and flaming process could be executed for more times. In our research, the mass of Co_3_O_4_-CNTs loaded on NF were about 0.1, 0.2, 0.4, 0.9, 1.5 mg, corresponding to the various immersing and flaming times (1, 2, 5, 10, 15).

### 2.3. The Synthesis of CoSe

CoSe particles were deposited on Co_3_O_4_-CNTs via electrodeposition process. The bath for electrodeposition consists of 25 mL DI water, 0.2 mmol CoCl_2_•6H_2_O, 0.2 mmol SeO_2_ and 200 mg LiCl•H_2_O. After being stirred evenly, a homogeneous pink solution was obtained. Through a typical three-electrode system on an electrochemical workstation (CHI 660C) (CH Instruments, Inc., Shanghai, China), the constant current electrodeposition procedure was realized by using Co_3_O_4_-CNTs/NF as working electrode, Pt foil and saturated calomel electrode (SCE) as the counter electrode and the reference electrode, respectively. The electrodeposition was conducted at 15 mA cm*^−^*^2^ for 1200 s. Pure CoSe nanoparticles were also deposited on NF without the flame process for comparison. The mass of CoSe/Co_3_O_4_-CNTs, Co_3_O_4_-CNTs and CoSe loaded on NF were 0.7, 0.4 and 1 mg, respectively.

### 2.4. Characterization

A Rigaku Miniflex600 X-ray diffraction (XRD) system (Rigaku, Tokyo, Japan) with Cu *K*_α_ irradiation was applied to analyze the crystal structures of samples. The chemical state of the synthetic elements was determined by X-ray photoelectron spectrometer (Thermo Scientific K-Alpha+) (Thermo Fisher Scientific, Waltham, MA, USA). A FEI Verios 460 scanning electron microscopy (SEM) was used to observe the morphologies and microstructures. The Brunauer–Emmett–Teller (BET) (Kubo X1000) analysis (Beijing Builder Electronic Technology Co., Ltd., Beijing, China) was used to study the specific surface area and pore size distribution of the sample. Raman spectra were obtained with an excitation wavelength of 514 nm, using a Horiba Scientific LabRAM HR Evolution. (HORIBA Scientific, Kyoto, Japan).

### 2.5. Electrochemical Measurements

The three-electrode system in 3 M KOH was introduced to investigate the electrochemical properties of the as-synthesized materials on NF, where an Hg/HgO electrode and a Pt-wire were used as the reference electrode and the counter electrode, respectively. Cyclic voltammetry (CV) measurements were tested, ranging from 0 to 0.6 V at different sweep rates (5–100 mV s*^−^*^1^), while galvanostatic charge–discharge (GCD) was measured within the potential window of 0–0.5 V at different current densities (1–10 A g*^−^*^1^). Electrochemical impedance spectroscopy (EIS) measurement was performed at a frequency between 0.01 and 100 kHz, where the amplitude was set to be 5 mV. The ASCs were assembled in 3 M KOH with the CoSe/Co_3_O_4_-CNTs/NF (or CoSe/NF) electrode as the positive electrode and the AC electrode as the negative electrode. The cycling stability was investigated by a Neware battery test instrument (BST-3008) (Neware Technology Limited, Shenzhen, China) in a voltage range of 0–1.6 V at 2 A g*^−^*^1^.

## 3. Results and Discussion

### 3.1. Characterization of Materials

**Scheme 1:**
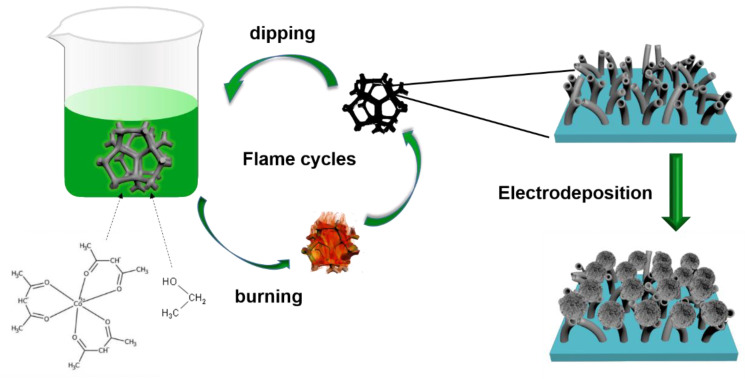
Schematic diagram of the process to prepare the CoSe/Co_3_O_4_-CNTs/NF electrode.

Scheme 1 illustrates the fabrication process of the CoSe/Co_3_O_4_-CNTs/NF electrode. The forming mechanism of Co_3_O_4_-CNTs is that the hydrocarbon of cobalt acetylacetonate are catalytic-decomposed to Co + C (Co_2_C, Co_3_C) over NF, then cobalt nanoparticles combined with oxygen atoms (air atmosphere) and Co_3_O_4_-CNTs is the final product [28,29,30]. Cobalt nanoparticles act as the catalyst for in situ formation of CNTs and oxidized to finally form Co_3_O_4_. The effect of different loading amounts on the morphology and properties of Co_3_O_4_-CNTs is shown in Figures S1 and S2. The SEM image with five flame cycles show the appropriate load amount. In Figure S2, the NF electrodes have a lower performance and almost no contribution to electrochemical performance. The Co_3_O_4_-CNTs electrodes with different flame cycles show extremely low capacitance (180 F g*^−^*^1^). After consideration, the Co_3_O_4_-CNTs/NF-0.4 (0.4 stans for the loading amount of 0.4 mg cm^−2^) was used for the next electrodeposition. In the process of cathodic current reduction, the SeO_2_ will be reduced stepwise to Se^2−^ and then reacted with the metal ions to form metal selenides. The electrodeposition mechanism can be interpreted by the following chemical equations [31,32]:

(1) $${\mathrm{SeO}}_{2}+4H^{+}+4e^{-}\to \mathrm{Se}+2H_{2}O$$

(2) $$\mathrm{Se}+2H^{+}+2e^{-}\to H_{2}\mathrm{Se}$$

(3) $${\mathrm{SeO}}_{2}+6H^{+}+6e^{-}\to H_{2}\mathrm{Se}+2H_{2}O$$

(4) $${\mathrm{Co}}^{2+}+{\;\mathrm{Se}}^{2-}\to \mathrm{CoS}e$$

**Figure 1:**
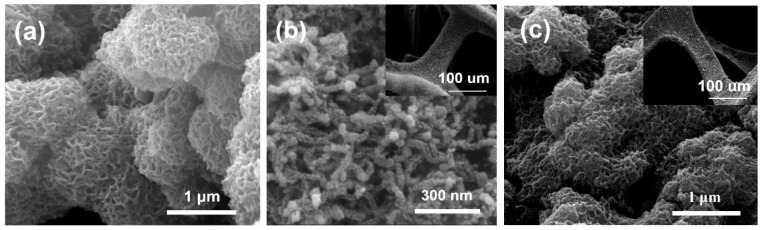
SEM images of (**a**) CoSe/NF, (**b**) Co_3_O_4_-CNTs/NF and (**c**) CoSe/Co_3_O_4_-CNTs/NF.

Figure 1 shows the SEM image of CoSe/NF, Co_3_O_4_-CNTs/NF and CoSe/Co_3_O_4_-CNTs/NF. The morphology of CoSe/NF with a homologous nanoparticle architecture with less pores is shown in Figure 1a,b, showing the typical SEM image of the Co_3_O_4_-CNTs/NF sample, indicating that Co_3_O_4_-CNTs coated on the surface of NF shows a loose structure with abundant micro-voids. This superhydrophilic architecture with excellent conductivity is believed to provide more nucleation sites for the subsequent electrodeposition to expand the specific surface area of composites, which is beneficial to promote the electrochemical properties of the electro-active materials [33,34]. XRD pattern of Co_3_O_4_-CNTs is shown in Figure S3. Three diffraction peaks of NF substrate (JCPDS no. 04-0850), which are located at 44.7°, 52.1° and 76.6°, can be seen clearly. The other four weak diffraction peaks at 31.4°, 36.9°, 59.6°, and 65.4° are assigned to Co_3_O_4_ (JCPDS no.74-1657). However, no diffraction peaks of CNTs are observed, which maybe imply the poor crystallinity of CNTs. The Raman spectrum in Figure S4 further indicated the relatively good graphitization of Co_3_O_4_-CNTs. The *I*_D_/*I*_G_ of about 0.96 suggests this carbon material possesses rich defects [35]. The TEM image (Figure S5) indicates the diameter of the CNTs is about 18 nm, and Co_3_O_4_(2–4 nm) nanoparticles adhere on the surface of CNTs and encapsulate at the tip. Figure 1c shows the SEM micrographs of the CoSe/Co_3_O_4_-CNTs/NF sample, which reveals a uniform nanoparticle-like architecture with plenty of pores on its surface. As mentioned above, this porous structure is beneficial for the infiltration of electrolyte and the improvement of electrochemical properties of CoSe. N_2_ adsorption–desorption technique was employed to analyze the characteristics of pores quantitatively. The specific surface area (SSA) and the pore diameter distribution of Co_3_O_4_-CNTs/NF, CoSe/NF and CoSe/Co_3_O_4_-CNTs/NF are displayed in Figure S6. As can be seen, all of them show type IV curves with mesoporous feature. The SSA of CoSe/Co_3_O_4_-CNTs/NF (77.4 m^2^ g*^−^*^1^) is much larger than that of CoSe/NF (17.1 m^2^ g*^−^*^1^) and Co_3_O_4_-CNTs/NF (21.5 m^2^ g*^−^*^1^). The insert patterns display Barrett–Joyner–Halenda (BJH) pore size distribution curves. Among them, CoSe/Co_3_O_4_-CNTs/NF displays the main pores of 3.21 nm in a range of 1–100 nm. The larger SSA and moderate mesoporous size feature contribute to the fast ion migration/diffusion during redox reaction [36]. Actually, double-layer capacitance (CDL) or Gaussian fitting of the SEM or TEM images method can also be used to analyze the pore diameter distributions, which were more convenient [37,38].

**Figure 2:**
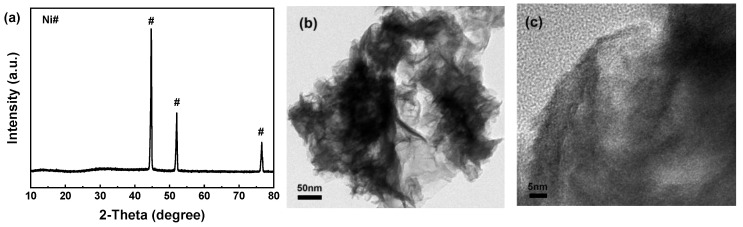
(**a**) XRD pattern of CoSe/Co_3_O_4_-CNTs/NF, (**b**,**c**) TEM images of CoSe stripped from the substrate.

Figure 2a depicts the representative XRD pattern of CoSe/Co_3_O_4_-CNTs/NF. As presented, three diffraction peaks at 44.7°, 52.1° and 76.5° can be assigned to the NF, while no other diffraction peaks can be observed, implying that the CoSe grown on the Co_3_O_4_-CNTs/NF mainly exists in amorphous form. The composition and chemical valence of the CoSe/Co_3_O_4_-CNTs/NF sample was further investigated by using XPS analysis (as shown in Figure S7). The full XPS survey spectrum of CoSe/Co_3_O_4_-CNTs/NF represents the co-existence of Co and Se elements (Figure S7a). Two major peaks at 795.92 and 780.48 eV in the high-resolution XPS spectrum of Co 2*p* (Figure S7b) correspond to the Co 2*p*_1/2_ and 2*p*_3/2_, with two satellites at 802.51 and 784.73 eV, respectively [34,39,40,41,42,43], which suggests the presence of Co^2+^. Figure S7c shows the detailed analysis of Se 3*d* high-resolution XPS spectra at a lower energy. Although there is interference on the Co 3*p* line on 59.1 eV, the emerged peaks at 55.8 and 54.8 eV can be appointed to the Se 3*d*_3/2_ and 3*d*_5/2_ [41,44], suggesting that the Se elemental mainly exists in Se^2^*^−^*. XPS results verify the successful formation of CoSe on the Co_3_O_4_-CNTs/NF substrate. In order to obtain the detailed morphology of samples, CoSe was stripped from the substrate via ultrasonic vibration slightly to avoid structural damage. The TEM image in Figure 2b shows that the CoSe displays a connected nanosheet-like structure construction with ultrathin thickness and a rough surface. Furthermore, no clear lattice fringes are observed in the corresponding high-resolution TEM pattern (Figure 2c), which means that the as-synthesized CoSe possesses a low-crystalline characteristic. This result is consistent with the result of XRD. Active materials with higher SSA and low-crystalline have been proven to be beneficial to provide more active sites and withstand the distortion of structure during the charge/discharge process [45], resulting in a higher specific capacitance and longer cycling stability. Therefore, we deduced that the as-synthesized CoSe/Co_3_O_4_-CNTs/NF will exhibit excellent electrochemical properties.

### 3.2. Electrochemical Performances of CoSe/Co_3_O_4_-CNTs/NF

**Figure 3:**
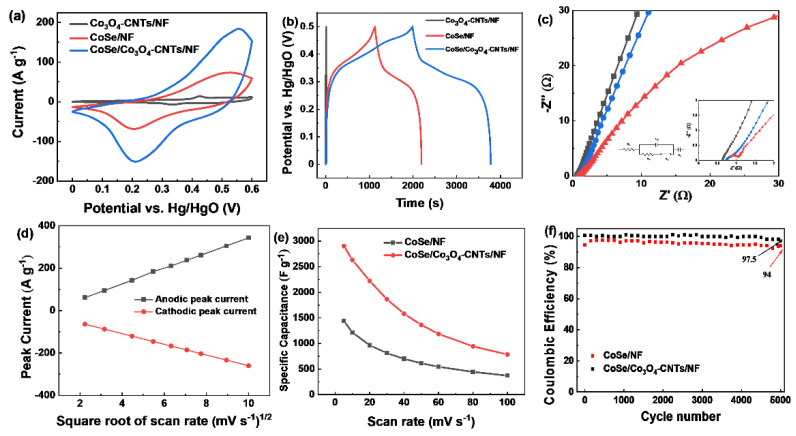
Electrochemical performances of Co_3_O_4_-CNTs/NF, CoSe/NF and CoSe/Co_3_O_4_-CNTs/NF: (**a**) CV curves scanned at a scan rate of 30 mV s*^−^*^1^; (**b**) GCD curves at a current of 1 A g*^−^*^1^, (**c**) Nyquist plots; (**d**) fitting lines of peak current density for the square root of scan rates of CoSe/Co_3_O_4_-CNTs/NF; (**e**) specific capacitance of CoSe/NF and CoSe/Co_3_O_4_-CNTs/NF electrodes; (**f**) cycling stability for 5000 cycles at 10 A g*^−^*^1^.

Electrochemical performances of the CoSe/Co_3_O_4_-CNTs/NF electrodes are shown in Figure 3. For the CV curves in Figure 3a, a pair of redox peaks clearly show the faradaic pseudocapacitive characteristics of the battery-like electrode, corresponding to the following redox reactions [46,47]:

(5) $${\mathrm{Co}}_{3}O_{4}+{\mathrm{OH}}^{-}+H_{2}O↔3\mathrm{CoOOH}+e^{-}$$

(6) $$2\mathrm{CoS}e+2H_{2}O+O_{2}\;↔2\mathrm{Co}{\left(\mathrm{OH}\right)}_{2}+2\mathrm{Se}\;$$

(7) $$3\mathrm{Se}+6{\mathrm{OH}}^{-}↔2{\mathrm{Se}}^{2-}+{\mathrm{SeO}}_{3}^{2-}+3H_{2}O$$

(8) $$\mathrm{Co}{\left(\mathrm{OH}\right)}_{2}+{\mathrm{OH}}^{-}\;↔\mathrm{CoOOH}+H_{2}O+e^{-}$$

(9) $$\mathrm{CoOOH}+{\mathrm{OH}}^{-}\;↔{\;\mathrm{CoO}}_{2}+H_{2}O+e^{-}$$

The distinct peaks may be attributed to the redox features of Co^2+^/Co^3+^ and Co^3+^/Co^4+^. Additionally, the CoSe/Co_3_O_4_-CNTs/NF electrode has a much larger integrated area than the CoSe/NF electrode, indicating that the CoSe/Co_3_O_4_-CNTs/NF electrode will provide a satisfying energy storage ability. Figure 3b displays the compared GCD curves of CoSe/Co_3_O_4_-CNTs/NF, CoSe/NF and Co_3_O_4_-CNTs/NF electrodes at 1 A g*^−^*^1^. Apparently, the CoSe/Co_3_O_4_-CNTs/NF delivered a higher specific capacitance of 2906 F g*^−^*^1^, which was two times larger than that of the CoSe/NF electrode (1440.8 F g*^−^*^1^). Co_3_O_4_-CNTs/NF has a relatively high surface area (Figure S6), and its capacitance is 180 F g*^−^*^1^ at 1 A g*^−^*^1^. 

To evaluate the charge transfer rate of the hybrid electrode, EIS tests were conducted (Figure 3c). The intercepts at the real axis are associated with *R*_s_ (contributions of the ionic resistance of the electrolyte, intrinsic resistance, and contact resistance between the active material and the current collector). In addition, the semicircles represent *R*_ct_ (charge-transfer resistance) of electrode materials. The slopes of the impedance line in the low-frequency region denote W (Warburg impedance), which indicates the ion diffusion in the interface of electrode material/electrolyte. The corresponded fitting results of Nyquist plots (Table S1) reveal that CoSe/Co_3_O_4_-CNTs/NF has a lower charge-transfer resistance (*R*_ct_ = 0.89 Ω cm^2^) and solution resistance (*R*_s_ = 0.73 Ω cm^2^), indicating its superior charge mobility. We also calculate the resistances of Co_3_O_4_-CNTs/NF, CoSe/NF and CoSe/Co_3_O_4_-CNTs/NF electrodes from the voltage drop in GCD plots at the higher current densities (100 to 500 A g*^−^*^1^) (Figure S8 and Table S2). The electrode of CoSe/Co3O4-CNTs/NF has the lower resistance 1.542 Ω cm^2^. The outstanding impedance characteristics of CoSe/Co_3_O_4_-CNTs/NF arise from the synergetic effect of Co3O4-CNTs and CoSe materials, in which the Co_3_O_4_-CNTs winded with each other can provide good conductivity and stability. Meanwhile, the larger SSA and moderate mesoporous size of CoSe/Co_3_O_4_-CNTs/NF can offer excellent infiltration of the electrolyte [36].

The CV cures of the CoSe/Co_3_O_4_-CNTs/NF electrode at various scan rates are depicted in Figure S9a. Notably, with the increasing of scan rates, the anodic and cathodic peaks shift to higher and lower voltage, respectively, suggesting the increased internal diffusion resistance of the electrode [48]. Meanwhile, the perfect linear relationship of *i* and *v*^1/2^ (Figure 3d) indicates the redox reactions between electrolyte and electrode interface at the electrolyte/electrode interface is related to a quasi-reversible and diffusion control process [24].

The specific capacitance and rate capacity of the CoSe/Co_3_O_4_-CNTs/NF electrode are further investigated by GCD test at different current densities (Figure S9b and Figure 3e). Obviously, each GCD curve shows an apparent charge–discharge platform without obvious voltage drop, indicating the excellent pseudocapacitance properties and electronic conductivity of the CoSe/Co_3_O_4_-CNTs/NF electrode. Moreover, the CoSe/Co_3_O_4_-CNTs/NF electrode has a rate capability (46.8% specific capacitance retention from 5 to 50 mV s^−1^), which is higher than that of CoSe/NF electrode (42.5%) at the same condition (Figure 3e) and other Co_x_Se_y_-based electrodes, such as core-branch CoSe_2_, CoSe_2_/C, CoSe_2_ nanosheet arrays (Table S3) [34,49,50,51,52,53]. The cycling performance of the CoSe/Co_3_O_4_-CNTs/NF electrode was further investigated. As shown in Figure 3f, after cycling at 10 A g*^−^*^1^ for 5000 cycles, the CoSe/Co_3_O_4_-CNTs/NF electrode still retains 97.5% of its initial capacitance, suggesting the CoSe/Co_3_O_4_-CNTs/NF electrode possesses satisfied long-term cycling stability due to the synergistic effects between the good graphitization of Co_3_O_4_-CNTs and CoSe.

### 3.3. Electrochemical Performances of CoSe/Co_3_O_4_-CNTs/NF//AC ASC

**Figure 4:**
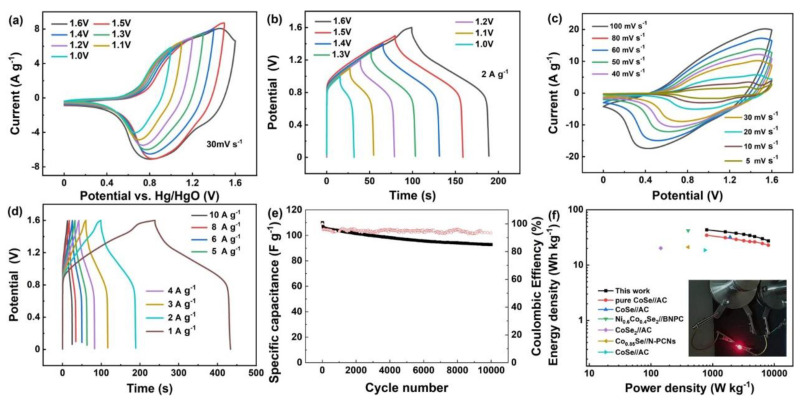
(**a**,**b**) CV and GCD curves of CoSe/Co_3_O_4_-CNTs/NF//AC ASC at different potential windows, (**c**,**d**) CV and GCD curves of CoSe/Co_3_O_4_-CNTs/NF//AC ASC at different scan rates and current densities, (**e**) cycling performance of CoSe/Co_3_O_4_-CNTs/NF//AC, (**f**) Ragone plot of CoSe/Co_3_O_4_-CNTs/NF//AC ASC. The inset in (**f**) is photographic images of the two series of connected CoSe/Co_3_O_4_-CNTs/NF//AC ASCs with a red LED.

To further explore the potential of CoSe/Co_3_O_4_-CNTs/NF composite electrodes for practical application, an ASC (CoSe/Co_3_O_4_-CNTs/NF//AC) was established in 3 M KOH using CoSe/Co_3_O_4_-CNTs/NF and AC as positive and negative electrodes, respectively. The mass ratio of AC and CoSe/Co_3_O_4_-CNTs in CoSe/Co_3_O_4_-CNTs/NF//AC ASC is 17.4:1, which was calculated by Formula (1) in the supporting information. At 10 mV s*^−^*^1^, the complementary CV curves of CoSe/Co_3_O_4_-CNTs/NF (2631.9 F g*^−^*^1^) and AC electrode (202.3 F g*^−^*^1^) in the typical three-electrode system were obtained in Figure S10, which inferred that the voltage window of CoSe/Co_3_O_4_-CNTs/NF//AC ASC can be extend to 1.6 V. Both the CV (Figure 4a) and GCD (Figure 4b) curves of CoSe/Co_3_O_4_-CNTs/NF//AC ASC at different voltage windows exhibit a good electrochemical stability, indicating that the CoSe/Co_3_O_4_-CNTs/NF//AC ASC can work stably at a high voltage window of 0 to 1.6 V or a low voltage window of 0 to 1.0 V.

Figure 4c reveals the CV curves of the CoSe/Co_3_O_4_-CNTs/NF//AC ASC measured at different scanning rates of 5–100 mV s*^−^*^1^. The shape of CV curves performs no obvious deformation with the increment of scan rates, verifying its good reversibility. As shown in Figure 4d, the near symmetry of GCD curves of the CoSe/Co_3_O_4_-CNTs/NF//AC ASC from 1 to 10 A g*^−^*^1^ confirm the good columbic efficiency (CE) of the device. At the different current density of 1, 2, 3, 4, 5, 6, 8 and 10 A g*^−^*^1^, the specific capacitances of CoSe/Co_3_O_4_-CNTs/NF//AC ASC are 121.9, 112.3, 105.9, 101, 96.6, 92.3, 85.5 and 76.9 F g*^−^*^1^. Figure S11 displays the compared GCD curves of the CoSe/Co_3_O_4_-CNTs/NF//AC and CoSe/NF//AC at 2 A g*^−^*^1^. The absolutely longer discharge time of CoSe/Co_3_O_4_-CNTs/NF//AC indicates its enhanced energy storage capability. In addition, CoSe/Co_3_O_4_-CNTs/NF//AC exhibit an excellent durableness with 92.7% capacitance retention after 10,000 cycles (Figure 4e), suggesting its practicability in real life time.

Furthermore, the Ragone plot in Figure 4f shows the functional relationship between the energy density and the power density of the ASC device, and the value could be obtained by Formulas (3) and (4) in the supporting information. A high energy density of 43.4 Wh kg^−1^ was obtained at 800 W kg*^−^*^1^, which is higher than that of CoSe/NF//AC (34.6 Wh kg*^−^*^1^ at 800 W kg^−1^) and many ASCs reported previous, such as CoSe_2_//AC [54], Ni_0.6_Co_0.4_Se_2_//BNPC [34], CoSe_2_//AC [49], Co_0.85_Se//N-PCNs [42] and CoSe//AC [40]. Additionally, a red LED bulb can be powered brightly by two CoSe/Co_3_O_4_-CNTs/NF//AC ASC devices in series for 15 min, suggesting the potential application of the ASC in energy storages (Figure 4f).

## 4. Conclusions

In summary, a free-standing and highly active CoSe/Co_3_O_4_-CNTs hybrid material was synthesized by electrodepositing CoSe nanoparticles on a forest of density and uniform Co_3_O_4_-CNTs supported by NF, which was used as a positive electrode of supercapacitor directly. Due to the synergist effects of the multicomponent, nanoporous structure and the enlarged SSA of the CoSe/Co_3_O_4_-CNTs composite material, the hybrid electrode delivered a high specific capacitance of 2906 F g*^−^*^1^, at 5 mV s*^−^*^1^ and 1362.8 F g*^−^*^1^, at 50 mV s*^−^*^1^. ACS assembled using CoSe/Co_3_O_4_-CNTs/NF and AC as positive and negative electrodes achieved a high energy density of 43.4 Wh kg*^−^*^1^ at 0.8 kW kg*^−^*^1^ with an excellent capacitance retention of 92.7% after 10,000 cycles. Meanwhile, it is worth noting that, due to the facile fabrication process, the novel hybrid electrode structure can be scaled up by supercapacitor manufacturers easily. This work presents an effective strategy to design a hybrid composite electrode for a high-performance asymmetric supercapacitor.

## Supplementary Materials

The following supporting information can be downloaded at: https://www.mdpi.com/article/10.3390/ma15175841/s1. Figure S1. SEM images of Co_3_O_4_-CNTs/NF with (a,b) 1, (c,d) 2, (e,f) 5, (g,h) 10 and (i,j) 15 flame cycles. Figure S2. Electrochemical properties of Co_3_O_4_-CNTs/NF electrode. (a) CV curves at 50 mV s^−1^, (b) GCD curves at 5 A g^−1^. Figure S3. XRD pattern of Co_3_O_4_-CNTs/NF. Figure S4. Raman spectra of Co_3_O_4_-CNTs/NF. Figure S5. TEM image of Co_3_O_4_-CNTs stripped from NF. Figure S6. N_2_ adsorption-desorption plots of (a) Co_3_O_4_-CNTs, (b) CoSe and (c) CoSe/Co_3_O_4_-CNTs. The inserts are corresponding pore diameter distribute. Figure S7. XPS spectra of CoSe/CNTs. (a) Survey spectrum and (b) Co *2p* and (c) Se *3d* spectra. Figure S8. The voltage drops in GCD curve at high current densities. Figure S9. Electrochemical properties of CoSe/Co_3_O_4_-CNTs/NF electrode. (a) CV curves at different scan rates, (b) GCD curves at different current densities. Figure S10. CV curves of the CoSe/Co_3_O_4_-CNTs/NF positive electrode and AC negative electrode scanned at 10 mV s^−1^. Figure S11. Comparison of GCD curves of CoSe/NF//AC and CoSe/Co_3_O_4_-CNTs/NF//AC ASCs at a current density of 2 A g^−1^. Table S1. The specific capacitance (Cs) of electrodes and the fitted parameters from the Nyquist plots in Figure 4c. Table S2. The resistances of Co_3_O_4_-CNTs/NF, CoSe/NF and CoSe/Co_3_O_4_-CNTs/NF. Table S3. Comparison of specific capacitance and rate capability with other related Cobalt Selenide electrode materials. Reference [54] is cited in the supplementary materials.
