# Supplementary material for: Facile Synthesis of CoSe/Co3O4-CNTs/NF Composite Electrode for High-Performance Asymmetric Supercapacitor
Source: Materials (Basel). 2022 Aug 24;15(17):5841. doi: 10.3390/ma15175841 (PMC9457315; doi:10.3390/ma15175841)
Supplement: Supplementary file 1 [file materials-15-05841-s001.zip › materials-1860754-supplementary.pdf]

*Supporting Information*

# Facile Synthesis of $CoSe/Co_3O_4$-CNTs/NF Composite Electrode for High-Performance Asymmetric Supercapacitor

**Ying Wang [1,2], Xiang Zheng [1,3], Xianjun Cao [3], Chengtao Yang [1], Qiang Zhao [1,3,*], Yongqi Zhang [1,4,*] and Xinhui Xia [1]**

[1] Yangtze Delta Region Institute (Huzhou), University of Electronic Science and Technology of China, Huzhou 313001, China
[2] School of New Energy and Materials, Southwest Petroleum University, Chengdu 610500, China
[3] School of Materials and Energy, University of Electronic Science and Technology of China, Chengdu 610054, China
[4] Institute of Fundamental and Frontier Sciences, University of Electronic Science and Technology of China, Chengdu 610054, China
***** Correspondence: zqphys@uestc.edu.cn (Q.Z.); yqzhang@uestc.edu.cn (Y.Z.)

**Keywords:** $CoSe/Co_3O_4$-CNTs composite electrode; flame method; ultra-high specific capacitance; long cycle life; asymmetric supercapacitor

The mass ratio of the CoSe/CNTs (or CoSe) electrode （$m_+$） and AC electrode （$m_-$） were rationally estimated by the following formulas [1–3]:

$$\frac{m_+}{m_-} = \frac{C_{s-}\Delta V_-}{C_{s+}\Delta V_+} \tag{S1}$$

Where $m$ is the mass, $C_s$ is the specific capacitance, and $\Delta V$ is the voltage range for positive (+) and negative (-) electrodes, respectively. The typical mass loading of an asymmetric supercapacitor is about 7 mg $cm^{-2}$. The specific capacitance ($C_s$, F $g^{-1}$) of the electrode materials was established by the following formula:

$$C_s = \frac{1}{mv\Delta V}\int_{V0}^{V} I(V)dV \tag{S2}$$

$$C_s = \frac{I\Delta t}{m\Delta V} \tag{S3}$$

Where $I$, $\Delta t$, m $v$, and $\Delta V$ are the discharge current (A), the discharge time (s), the mass of active material (g), the scan rate (V $s^{-1}$) and the discharge potential window (V), respectively.

Energy density is a significant condition to evaluate the practical application of the asymmetric supercapacitor. The energy density ($E$, Wh $kg^{-1}$) and power density ($P$, W $kg^{-1}$) of ASC could be calculated according to the following formulas:

$$E = \frac{C_s\Delta V^2}{7.2} \tag{S4}$$

$$P = \frac{3600E}{\Delta t} \tag{S5}$$

Where $C_s$, $\Delta V$ and $\Delta t$ are the specific capacitance, the potential window and the discharge time, respectively.

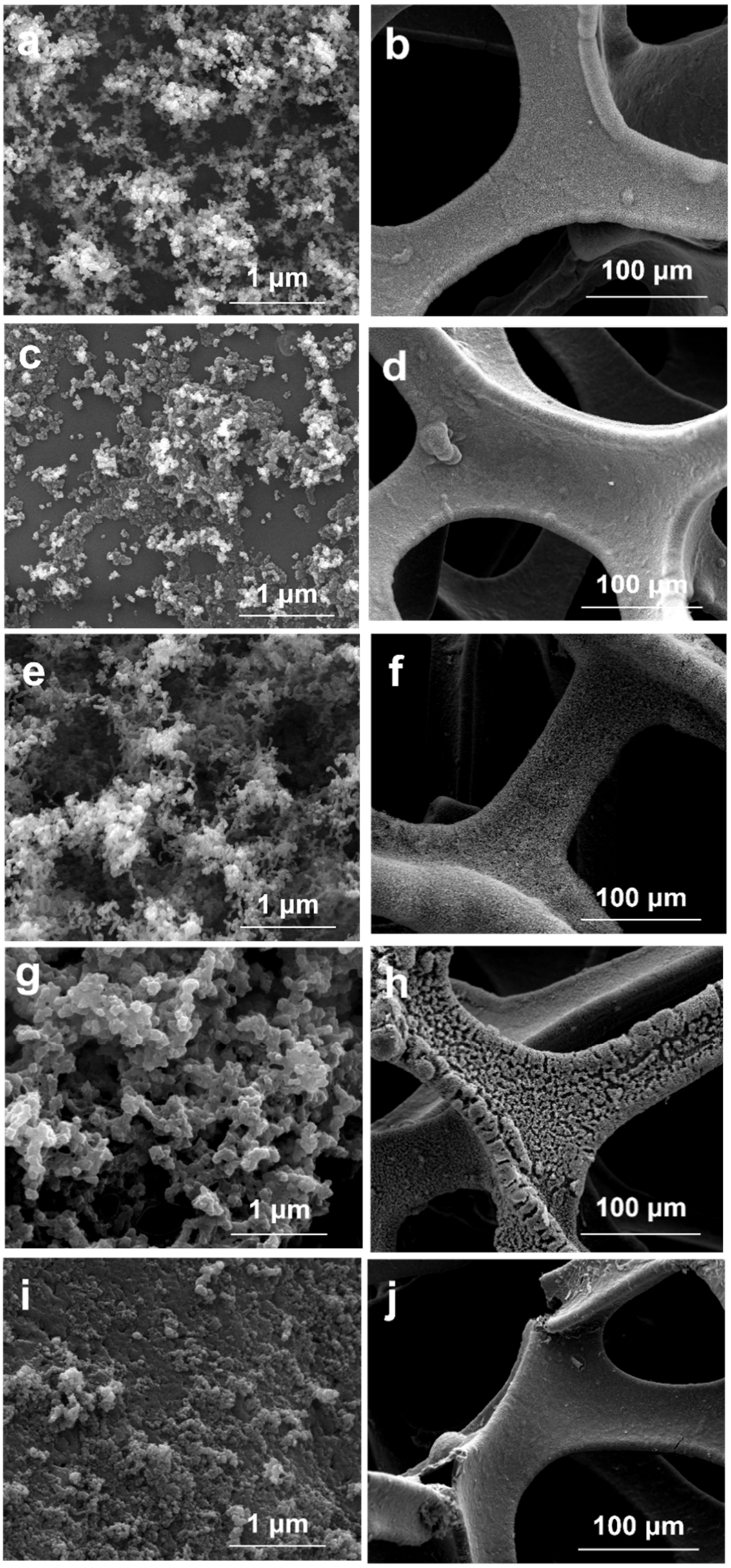


**Figure S1.** SEM images of $Co_3O_4$-CNTs/NF with (**a**,**b**) 1, (**c**,**d**) 2, (**e**,**f**) 5, (**g**,**h**) 10 and (**i**,**j**) 15 flame cycles.

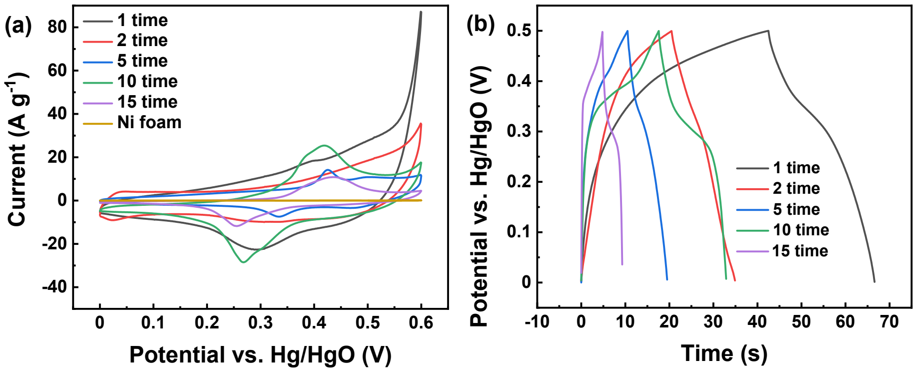


**Figure S2.** Electrochemical properties of $Co_3O_4$-CNTs/NF electrode. (**a**) CV curves at 50 mV $s^{-1}$, (**b**) GCD curves at 5 A $g^{-1}$.

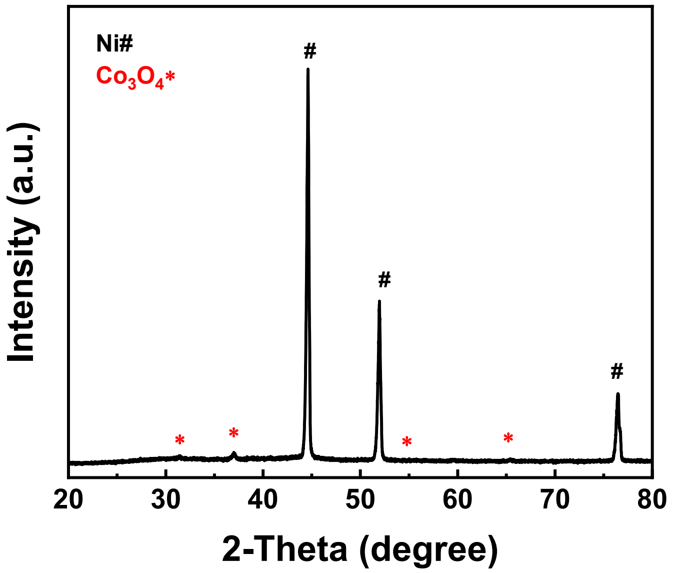


**Figure S3.** XRD pattern of $Co_3O_4$-CNTs/NF.

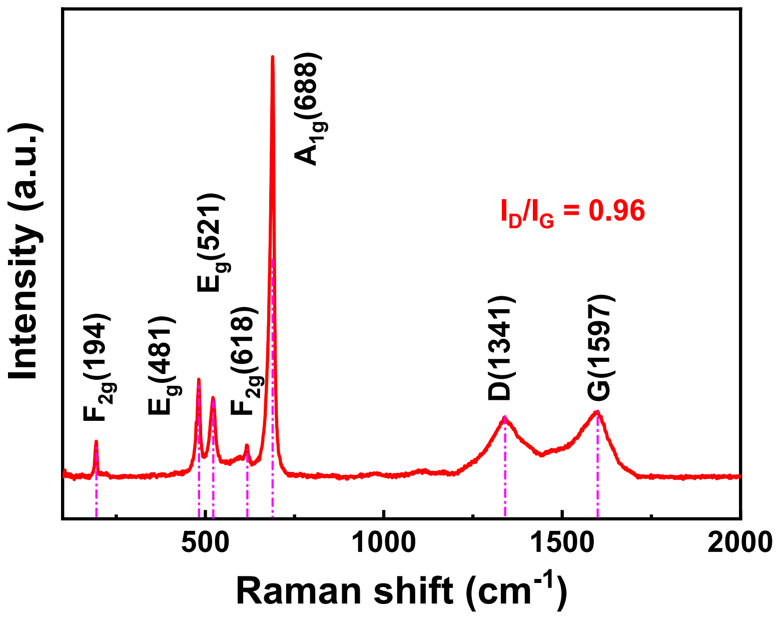


**Figure S4.** Raman spectra of $Co_3O_4$-CNTs/NF.

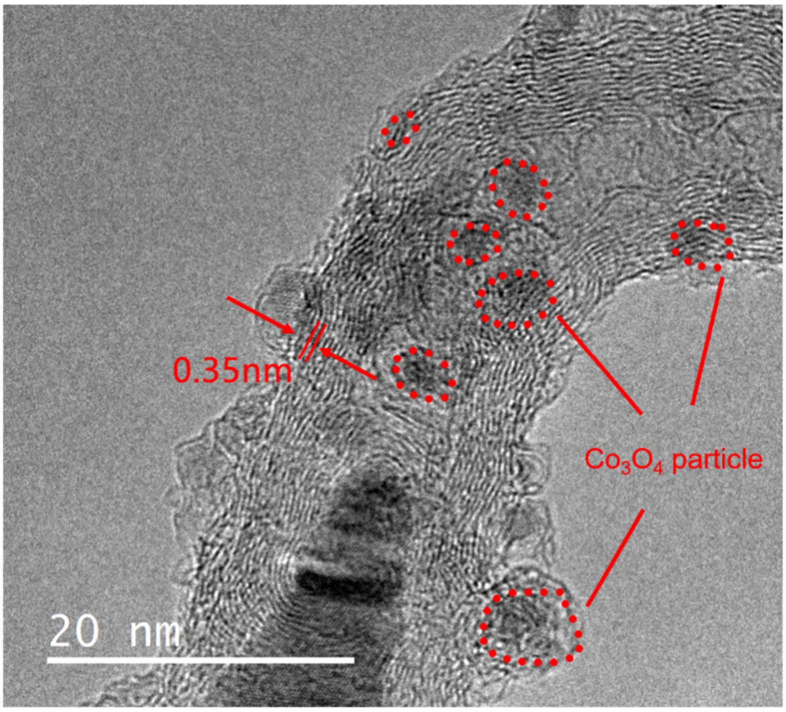


**Figure S5.** TEM image of $Co_3O_4$-CNTs stripped from NF.

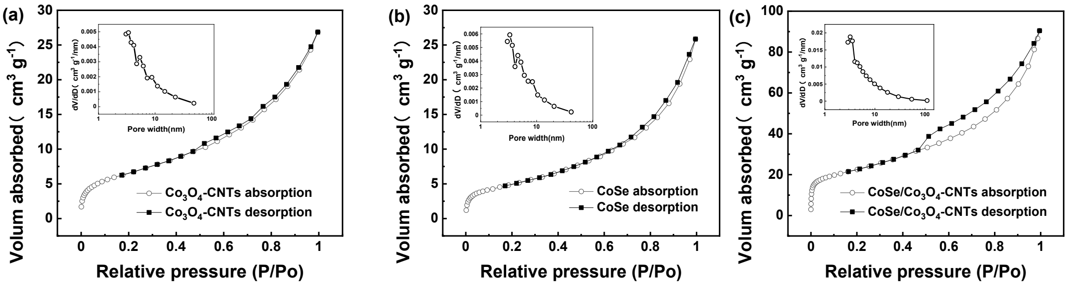


**Figure S6.** $N_2$ adsorption-desorption plots of (**a**) $Co_3O_4$-CNTs, (**b**) CoSe and (**c**) CoSe/$Co_3O_4$-CNTs. The inserts are corresponding pore diameter distribute.

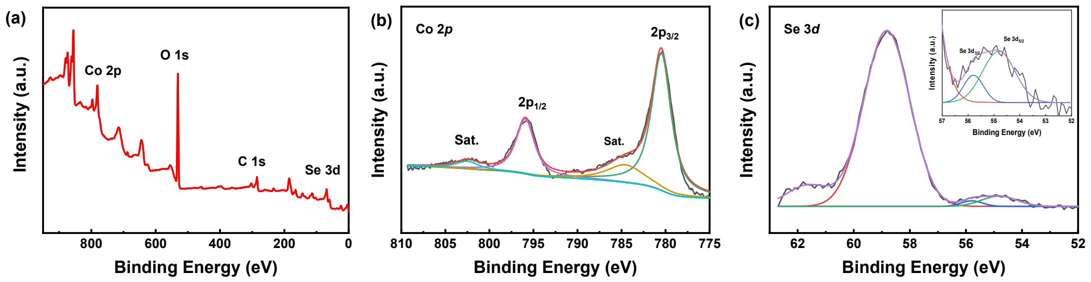


**Figure S7.** XPS spectra of CoSe/CNTs. (**a**) Survey spectrum and (**b**) Co *2p* and (**c**) Se *3d* spectra.

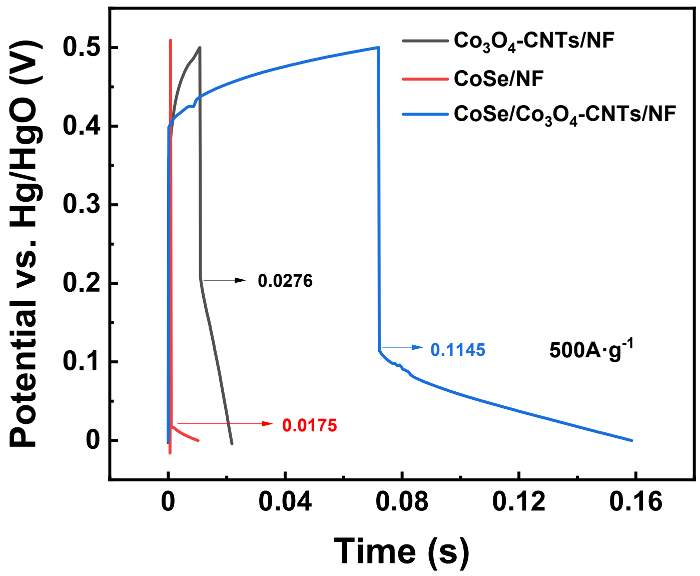


**Figure S8.** The voltage drops in GCD curve at high current densities.

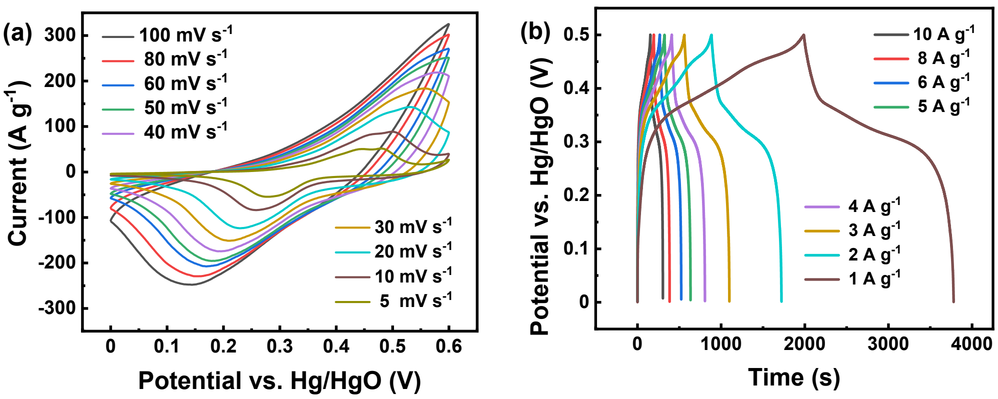


**Figure S9.** Electrochemical properties of CoSe/$Co_3O_4$-CNTs/NF electrode. (**a**) CV curves at different scan rates, (**b**) GCD curves at different current densities.

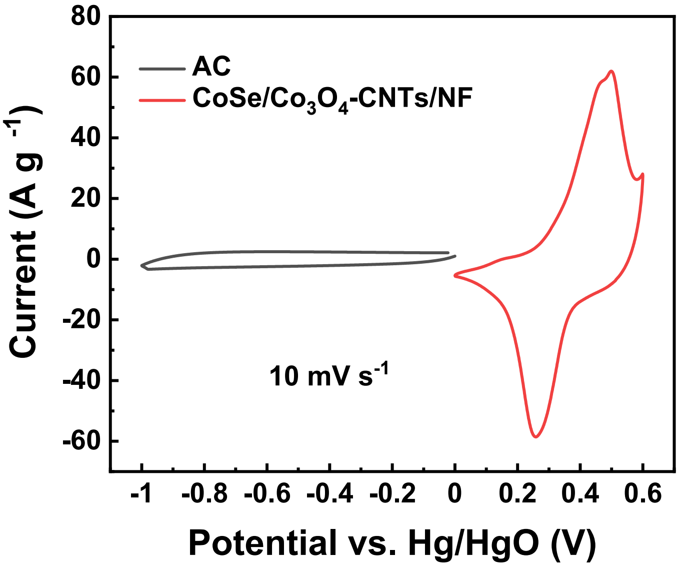


**Figure S10.** CV curves of the CoSe/$Co_3O_4$-CNTs/NF positive electrode and AC negative electrode scanned at 10 mV $s^{-1}$.

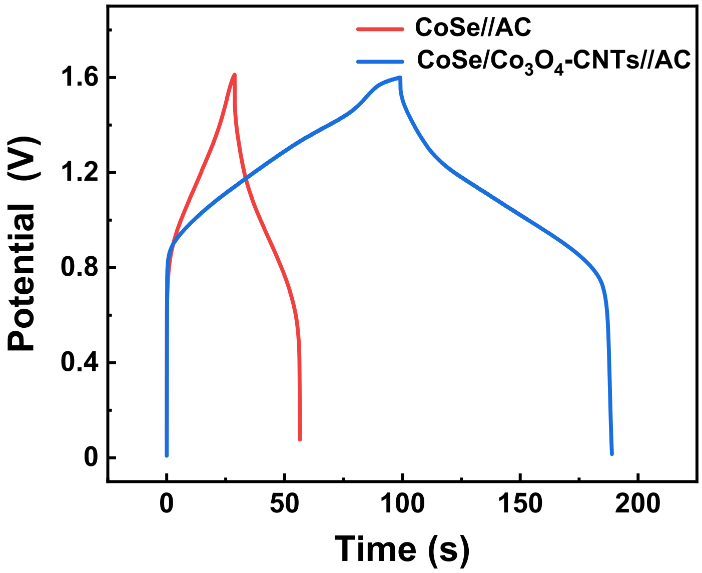


**Figure S11.** Comparison of GCD curves of CoSe/NF//AC and CoSe/$Co_3O_4$-CNTs/NF//AC ASCs at a current density of 2 A $g^{-1}$.

**Table S1.** The specific capacitance (Cs) of electrodes and the fitted parameters from the Nyquist plots in Figure 4c

| Sample | Cs (F $g^{-1}$) | Rs（Ω $cm^2$） | CPE, Yo (S $s^n$) | Freq power. n [0<n<1] | Rct（Ω $cm^2$） | W, Yo (S $s^{1/2}$ $cm^{-2}$) |
|---|---|---|---|---|---|---|
| CoSe/$Co_3O_4$-CNTs/NF | 2906 | 0.73 | 0.02 | 0.75 | 0.89 | $1\times10^{-3}$ |
| $Co_3O_4$-CNTs/NF | 180 | 0.64 | 0.0043 | 0.79 | 0.65 | $2.4\times10^{-14}$ |
| CoSe/NF | 1440.8 | 0.83 | 0.0039 | 0.67 | 1.042 | $2.8\times10^{-4}$ |

**Table S2.** The resistances of $Co_3O_4$-CNTs/NF, CoSe/NF and CoSe/$Co_3O_4$-CNTs/NF.

| Current density ($A\ g^{-1}$) / Resistance($\Omega\ m^2$) | 500 | 400 | 300 | 200 | 100 |
|---|---|---|---|---|---|
| $Co_3O_4$-CNTs/NF | 1.949 | 1.887 | 1.817 | 1.76 | 1.717 |
| CoSe/NF | 1.967 | 1.851 | 1.813 | 1.78 | 1.776 |
| CoSe/$Co_3O_4$-CNTs/NF | 1.542 | 1.53 | 1.505 | 1.508 | 1.446 |

**Table S3.** Comparison of specific capacitance and rate capability with other related Cobalt Selenide electrode materials.

| Materials | Capacitance | Rate capability | Reference |
|---|---|---|---|
| Core-Branch $CoSe_2$ | 759.5 F $g^{-1}$ at 1 mA $cm^{-2}$ | 67.1%(1-20 mA $cm^{-2}$) | [4] |
| $CoSe_2$ Nanosheet Arrays | 713.9 F $g^{-1}$ at 1 mA $cm^{-2}$ | 75.1%(1-20 mA $cm^{-2}$) | [5] |
| $CoSe_2$/C | 726 F $g^{-1}$ at 2A $g^{-1}$ | 62.7%(2-50 A $g^{-1}$) | [6] |
| $Ni_{0.6}Co_{0.4}Se_2$ | 602.6 C $g^{-1}$ at 1 A $g^{-1}$ | 77.7%(1-20 A $g^{-1}$) | [7] |
| V–$CoSe_2$ | 1830.2 F $g^{-1}$ at 1.5A $g^{-1}$ | 74.6%(1.5-2.5 A $g^{-1}$) | [8] |
| $Ni_{0.67}Co_{0.33}Se$ | 535 C $g^{-1}$ at 1 A $g^{-1}$ | 58.5%(1-50 A $g^{-1}$) | [9] |
| CoSe/$Co_3O_4$-CNTs | 2906 F $g^{-1}$ at 5 mV $s^{-1}$ | 46.8%(5-50-mV $s^{-1}$) | This work |

## Reference


1. Zheng, Y.Y.; Tian, Y.R.; Sarwar, S.; Luo, J.J.; Zhang, X.Y., Carbon nanotubes decorated NiSe2 nanosheets for high-performance supercapacitors. *J. Power Sources* **2020**, *452*, 8.
2. Zhao, L.C.; Zhang, P.; Zhang, Y.N.; Zhang, Z.; Yang, L.; Chen, Z.G., Facile synthesis of hierarchical Ni3Se2 nanodendrite arrays for supercapacitors. *J. Mater. Sci. Technol.* **2020**, *54*, 69–76.
3. Wang, X.H.; Fang, Y.; Shi, B.; Huang, F.F.; Rong, F.; Que, R.H.; Shao, M.W., Fabrication of Isomorphous Co3O4@Co3O4 Hierarchical Core-Shell Nanoneedles for High-Performance Supercapacitors. *ChemistrySelect* **2017**, *2*, 9267–9276.
4. Chen, T.; Li, S.Z.; Gui, P.B.; Wen, J.; Fu, X.M.; Fang, G.J., Bifunctional bamboo-like CoSe2 arrays for high-performance asymmetric supercapacitor and electrocatalytic oxygen evolution. *Nanotechnology* **2018**, *29*, 10.
5. Chen, T.; Li, S.Z.; Wen, J.; Gui, P.B.; Fang, G.J., Metal-Organic Framework Template Derived Porous CoSe2 Nanosheet Arrays for Energy Conversion and Storage. *ACS Appl. Mater. Interfaces* **2017**, *9*, 35927–35935.
6. Zhang, Y.F.; Pan, A.Q.; Wang, Y.P.; Cao, X.X.; Zhou, Z.L.; Zhu, T.; Liang, S.Q.; Cao, G.Z., Self-templated synthesis of N-doped CoSe2/C double-shelled dodecahedra for high-performance supercapacitors. *Energy Storage Mater.* **2017**, *8*, 28–34.
7. Wang, Y.H.; Liu, R.N.; Sun, S.X.; Wu, X.L., Facile synthesis of nickel-cobalt selenide nanoparticles as battery-type electrode for all-solid-state asymmetric supercapacitors. *J. Colloid Interface Sci.* **2019**, *549*, 16–21.
8. Sakthivel, M.; Ramaraj, S.; Chen, S.M.; Ho, K.C., Bimetallic vanadium cobalt diselenide nanosheets with additional active sites for excellent asymmetric pseudocapacitive performance: Comparing the electrochemical performances withM-CoSe2 (M = Zn, Mn, and Cu). *J. Mater. Chem. A* **2019**, *7*, 12565–12581.
9. Chen, H.C.; Chen, S.; Fan, M.D.; Li, C.; Chen, D.; Tian, G.L.; Shu, K.Y., Bimetallic nickel cobalt selenides: A new kind of electroactive material for high-power energy storage. *J. Mater. Chem. A* **2015**, *3*, 23653–23659.
